# The Chemical and Genetic Characteristics of Szechuan Pepper (*Zanthoxylum bungeanum* and *Z. armatum*) Cultivars and Their Suitable Habitat

**DOI:** 10.3389/fpls.2016.00467

**Published:** 2016-04-19

**Authors:** Li Xiang, Yue Liu, Caixiang Xie, Xiwen Li, Yadong Yu, Meng Ye, Shilin Chen

**Affiliations:** ^1^Key Laboratory of Beijing for Identification and Safety Evaluation of Chinese Medicine, Institute of Chinese Materia Medica, China Academy of Chinese Medical SciencesBeijing, China; ^2^Institute of Medicinal Plant Development, Chinese Academy of Medical Sciences and Peking Union Medical CollegeBeijing, China; ^3^College of Forestry, Sichuan Agricultural UniversityYa'an, China

**Keywords:** Szechuan pepper, *Zanthoxylum*, volatile oil, non-volatile ether extract, ecological factors, suitable habitat

## Abstract

Szechuan peppers, famous for their unique sensation and flavor, are widely used as a food additive and traditional herbal medicine. *Zanthoxylum bungeanum* and *Z. armatum* are both commonly recognized as Szechuan peppers, but they have different tastes and effects. The chemical components, genetic characteristics, and suitable habitat of six cultivars were analyzed in this study. The results indicated that *Z. armatum* contained a larger proportion of volatile oil, whereas *Z. bungeanum* produced a more non-volatile ether extraction. The average content of volatile oil and non-volatile ether extract of *Z. armatum* were 11.84 and 11.63%, respectively, and the average content of volatile oil and non-volatile ether extract of *Z. bungeanum* were 6.46 and 14.23%, respectively. Combined with an internal transcribed spacer 2 (ITS2) sequence characters and chemical PCA results, six cultivars were classified into their own groups, for the two species in particular. The temperature in January and July were the most significant ecological factors influencing the contents of the *Z. armatum* volatile oil. However, annual precipitation, temperature in January and relevant humidity had a significant positive correlation with the content of non-volatile ether extract in *Z. bungeanum*. Thus, the most suitable areas for producing *Z. bungeanum* cultivars ranged from the Hengduan Mountains to the Ta-pa Mountains, and the regions suitable for *Z. armatum* cultivars were found to be in the Sichuan Basin and Dalou-Wu mountains. The predicted suitable habitat could be used as a preliminary test area for Szechuan pepper cultivar production.

## Introduction

Szechuan peppers and their related species have been widely used in cooking and medical treatment in traditional Asian cultures, especially in China, where they have been used for centuries. Only the dried fruit with a special aroma, described as the “toothache tree,” has edible and medicinal value (Bautista et al., 2008). However, Szechuan peppers, widely consumed as a spice, are renowned as “magical” and one of “eight essential condiments” in the kitchen because of their exceptional aroma and flavor, with a slightly numbing effect like that from carbonated drinks. Szechuan peppers have also been applied to the treatment of pain, vomiting, diarrhea, ascariasis, and topical eczema treatment, owing to their anesthetic, stomachic, carminative and counter-irritant properties (State Pharmacopeia Committee, 2010; Brijwal et al., 2013; Singh et al., 2013). The special flavor of Szechuan peppers is mainly closely related to their chemical compounds, such as their volatile oil, alkaloids, acid amide, coumarin, lignin, fatty acid, and the small amount of triterpene and sterols that they contain. Among these components, volatile oil, alkaloids and acid amide are the main sources of fragrance and numbing effect (Xue and Pu, 2013). Furthermore, Szechuan peppers play an important role in the Conversion of Cropland to Forestland Program and in the transition of the rural industrial structure due to their ability to conserve soil and water, generate income and improve the livelihoods of local farmers (Zhang et al., 2005).

**Figure 1:**
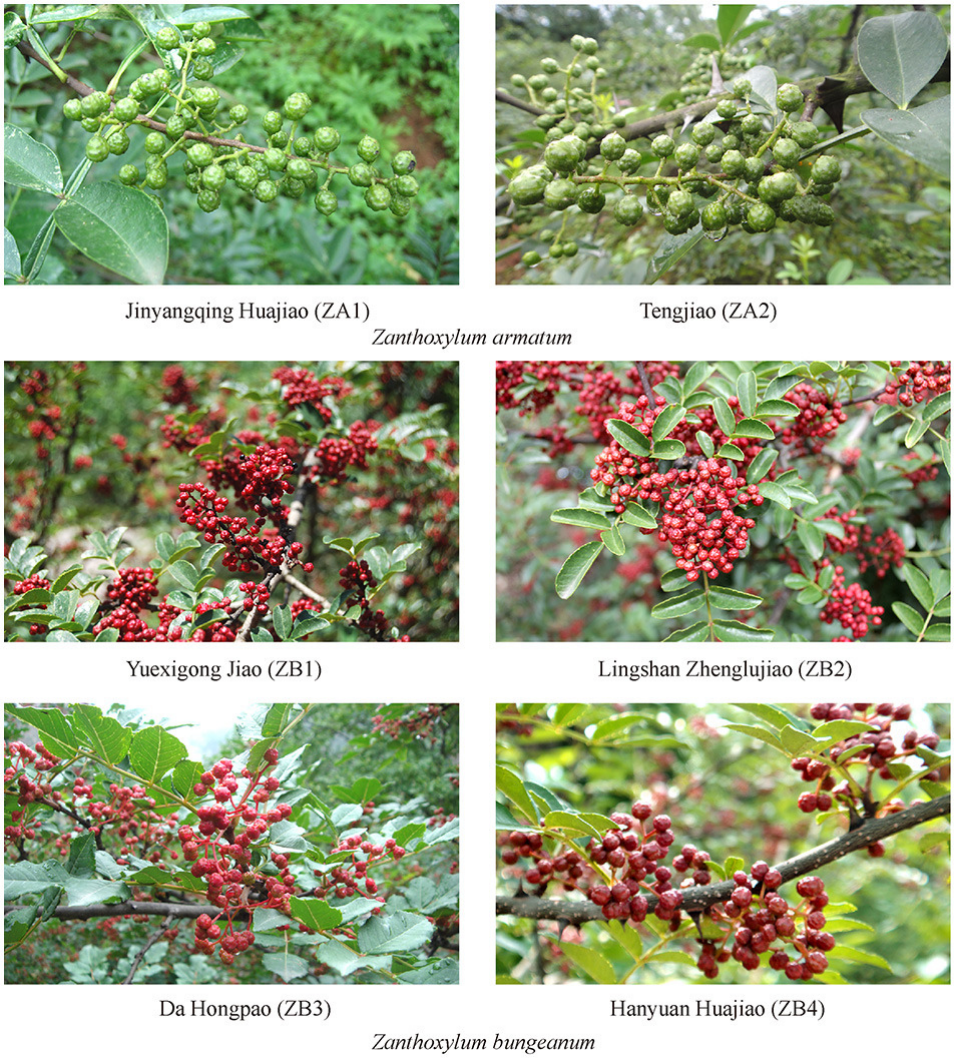
**Six quality cultivars of *Z. armatum* and *Z. bungeanum* during the mature period**. ZA1 and ZA2 belong to *Z. armatum* and the pericarps are bright green; ZB1-ZB4 belong to *Z. bungeanum* and the pericarps are bright red.

Chinese farmers have cultivated multiple cultivars of Szechuan peppers during the process, such as Yuexigong Jiao, Lingshan Zhenglujiao, Da Hongpao, and Hanyuan Huajiao, which belong to *Zanthoxylum bungeanum*. In recent years, Jinyangqing Huajiao and Tengjiao, which belong to *Z. armatum*, have also been cultivated. The pericarp color of *Z. armatum* cultivars is bright green and has a more unique aromatic smell; these cultivars are commonly known as “Qinghuajiao” (Figure 1). The pericarp color of *Z. bungeanum* cultivars are bright red, and they provide a more numbing sensation; these cultivars are commonly known as “Honghuajiao” (Figure 1; Yu et al., 2009). According to research, the cultivars of Szechuan peppers and their related plants are easily confused for their rich biodiversity and morphological similarity (Hou et al., 2014). However, the main chemical contents and suitable habitat are different between these cultivars. The incorrect selection of cultivars in agricultural production can cause economic loss.

This present study combined molecular and chemical techniques, Geographic Information Systems (GIS) and chemometrics analysis (Kress et al., 2005; Sucher and Carles, 2008; Shi et al., 2011; Song et al., 2012; Chen et al., 2014) (1) to assess the chemical characteristics of different Szechuan pepper cultivars, (2) to identify Szechuan pepper cultivars using combined chemical and molecular methods, and (3) to analyze the correlation between chemical contents and special ecological factors, which were then used to predict suitable habitat for Szechuan pepper cultivars.

## Materials and methods

### Determination of volatile oil (%) and non-volatile ether extract (%) content

A total of 47 batches (dry weight of 5000 g per batch) of six excellent cultivars of Szechuan peppers that belong to either *Z. armatum* or *Z. bungeanum* cultivated plants were collected during the mature period in 2014. The cultivars included Yuexigong Jiao (ZB1), Lingshan Zhenglujiao (ZB2), Da Hongpao (ZB3), and Hanyuan Huajiao (ZB4), which belong to *Z. bungeanum*, and Jinyangqing Huajiao (ZA1) and Tengjiao (ZA2), which belong to *Z. armatum* (Figure 1). The morphological features and cultivation practice information for six cultivars are listed in Supplementary Table S1 and on a seeding website (http://www.sclmzm.com:88/sczmz/linmuliangzhong.jhtml). The quality of the air, soil and water at all of the sample sites achieved the requirements of the Standard of Ambient Air Quality Standards (GB3095-2012), Soil Environment (GB15618-1995), and the Standards for irrigation water quality (GB 5084-2005), respectively.

#### Determination of volatile oil (%)

The volatile oil was evaluated according to the volatile oil determination method described in the Commerce Department Standard of the People's Republic of China (SB/T 10040-92) (Commerce Department Standard of the People's Republic of China, 1992) by a volatile oil determination device with steam distillation method. 20.00 g dried pericarp was accurately weighed in a 500 mL flask with three replications; 300 mL water and some glass beads were added, which were then connected to a volatile oil determination apparatus and a condenser pipe. Water was added from the top of the condenser pipe to a volatile oil determination apparatus over the scale until overflowing the flask. The water in flask was heated to boiling and maintained for 5 h until the volatile oil volume no longer increased. Stop heating and cooled the device in room temperature for a few minutes, open the piston of the volatile oil determination apparatus until the upper end of the oil dropped to 5 mm higher than the zero scale. After 1 h, open the piston again until the upper end of the oil dropped to the zero scale. Then read the volatile oil volume, and the content was calculated with the following formula:

T=V/M×100%

T: content of volatile oil (%)
V: volume of volatile oil (mL)
M: mass of the sample (g)

#### Determination of non-volatile ether extract (%)

The non-volatile ether extract was determined according to the State Standard of the People's Republic of China for spices and condiments determination for non-volatile ether extract (GB/T 12929.12-2008) (General Administration of Quality Supervision, Inspection and Quarantine of the People's Republic of China, Standardization Administration of the People's Republic of China, 2008). A 2.000 g sample was accurately weighed with three replications and encased with filter paper; it was then extracted by Soxhlet extraction with absolute ethyl ether for 18 h. The solvent was recovered to 1~2 mL, evaporated in a water bath and heated in an oven at 110°C for 1 h. The sample was then weighed after cooling in a dryer. The steps were repeated several times until two consecutive weights of less than 2 mg. The non-volatile ether extract content was calculated using the following formula:

$$\text{X}=({\text{m}}_{2}-{\text{m}}_{1})\times \frac{100}{{\text{m}}_{0}}\times \frac{100}{100-\text{H}}$$

X: content of non-volatile ether extract (%)
m_0_: mass of the sample
m_1_: mass of the receiving flask
m_2_: mass of the receiving flask and non-volatile ether extract (g)
H: water content of the sample (%)

After that, the volatile oil and non-volatile ether extract contents of Szechuan peppers were analyzed with a one-way analysis of variance (ANOVA) test (SPSS 19) and a principal component analysis (PCA) (SIMCA-P, version 11.5, Umetrics, Umeå, Sweden) (Xiao et al., 2011).

### Genetic diversity analysis

A nuclear ribosomal internal transcribed spacer 2 (ITS2) sequence possesses highly interspecific divergence and the capability to distinguish closely related taxa of medicinal plant at the species level (Chen et al., 2010). In this study, 119 specimens of Szechuan pepper cultivars and seven related species were sampled (Supplementary Table S2). One sequence of *Z. ovalifolium* var. *spinifolium* was also downloaded from the GenBank database. DNA extraction, PCR amplification and sequencing were performed as previously described (Chen et al., 2010; Xin et al., 2015). Consensus sequences and coting generation were performed using CondonCode Aligner V 3.7.1 (CodonCode Co., Centreville, MA, USA). All of the ITS2 sequences were annotated with the Hidden Markov Model (HMM) (Wolf et al., 2005; Keller et al., 2009). The sequences were aligned using Muscle, the genetic distance computed by MEGA 6.0 (Center for Evolutionary Medicine and Informatics, Tempe, Arizona, USA) based on the K2P model (Tamura et al., 2011). A phylogenetic tree constructed by employing the neighbor-joining (NJ) tree method, and bootstrap tests were conducted by applying 1000 resamples to assess the confidence in phylogenetic analysis using MEGA 6.0. Three sequences of *Toddalia asiatica* downloaded from GenBank were chosen as outgroup when the NJ tree was built.

### A correlation analysis of chemical composition contents and ecological factors and suitable habitat prediction

A regional information system of traditional Chinese medicine (RISTCM, number 2014SR159435), a GIS-based computer program, was self-developed for the spatial prediction of traditional Chinese medicinal plants (Institute of Chinese Materia Medica, China Academy of Chinese Medical Sciences, Institute of Medicinal Plant Development, Chinese Academy of Medical Sciences and Peking Union Medical College, 2014). Integrating national geographic, climate (1971–2000) and soil type databases in China, RISTCM was able to determine the impacts of environmental gradients and to predict the large-scale distribution of target medicinal plants (Huang et al., 2010; Wei et al., 2012; Xie et al., 2014b). RISTCM defines the native habitats of a target plant using fuzzy mathematics method based on specimen examination and extracts the ecological factors of native habitats from its databases. Combined with GPS coordinate data from the sample locations, 11 specific ecological factors that were closely related to the development of the crops in agriculture, including elevation (*x*1), active accumulated temperature (*x*2), sunshine duration (*x*3), mean annual temperature (*x*4), minimum temperature in January (*x*5), average temperature in January (*x*6), maximum temperature in July (*x*7), average temperature in July (*x*8), annual precipitation (*x*9), relative humidity (*x*10), and soil type (*x*11), were abstracted using RISTCM (Supplementary Table S3) (Chen et al., 2008; Yu et al., 2010; Xie et al., 2014a,b).

A correlation analysis of the main chemical compositions of the six Szechuan pepper cultivars and the corresponding ecological factors were performed using a partial least squares regression (PLSR) with VIP scores (SIMCA-P, V 11, Umetrics, Umeå, Sweden) (Seemann et al., 2010; Yang et al., 2011; Jia et al., 2012). Based on these ecological factors, the suitable habitat for *Z. bungeanum* and *Z. armatum* (cultivars) was then predicted based on ecological similarity using a grid-based spatial cluster analysis, vector-based overlaying, intersection analysis and an area calculation using RISTCM (Chefaoui et al., 2005; Liao, 2005; Chen et al., 2008; Yu et al., 2010).

## Results

### Analysis of chemical composition

A comparison of volatile oil with non-volatile ether extract contents signified that the average content of the volatile oil of *Z. armatum* was higher than that of *Z. bungeanum* but was not same as the non-volatile ether extract of *Z. armatum* (Table 1). The average content of volatile oil and non-volatile ether extract in *Z. armatum* were 11.84 and 11.63%, respectively, and the contents in *Z. bungeanum* were 6.46 and 14.23%, respectively. ZA2 (12.78%) contained the most volatile oil, and ZB1 (15.55%) contained the most non-volatile ether extract. A one-way ANOVA indicated that the two species were significantly different in their content of volatile oil and non-volatile ether extract (*P* < 0.05), and each cultivar was also significantly different (*P* < 0.05).

**Table 1:** **The locality and content of volatile oil and non-volatile ether extract from different Szechuan pepper cultivars**.

**Species**	**Cultivars**	**Abbreviation**	**Locality**	**Longitude**	**Latitude**	**Average content of volatile oil/%**	**Average content of non-volatile ether extracted/%**
*Z. armatum*	Jinyangqing Huajiao	ZA1	Ribu Village Taoping Town Jinyang County Sichuan Province China	103°15′22.372″	27°38′39.763″	7.49	9.77
	Jinyangqing Huajiao	ZA1	Luxiang Village Honglian Town Jinyang County Sichuan Province China	103°9′3.753″	27°35′13.75″	14.16	16.29
	Jinyangqing Huajiao	ZA1	Guangming Village Pailai Town Jinyang County Sichuan Province China	103°8′7.217″	27°34′44.197″	9.99	12.29
	Jinyangqing Huajiao	ZA1	Qingsong Village Jinyang County Sichuan Province China	103°8′8.287″	27°35′20.817″	12.91	11.07
	Tengjiao	ZA2	Zhige Town Hongya County Sichuan Province China	103°15′5.026″	29°55′.564″	10.00	10.12
	Tengjiao	ZA2	Wulong Village Zhige Town Hongya County Sichuan Province China	103°14′30.762″	29°54′55.425″	15.00	11.47
	Tengjiao	ZA2	Doudan Village Beijiao Town Ya'an City Sichuan Province China	102°58′59.941″	29°59′12.439″	13.33	10.39
*Z. bungeanum*	Yuexigong Jiao	ZB1	Qingsong Village Banqiao Town Yuexi County Sichuan Province China	102°33′32.35″	28°45′20″	6.67	17.07
	Yuexigong Jiao	ZB1	Qingsong Village Banqiao Town Yuexi County Sichuan Province China	102°33′32.36″	28°45′21″	7.47	16.92
	Yuexigong Jiao	ZB1	Qingsong Village Banqiao Town Yuexi County Sichuan Province China	102°33′34.42″	28°45′25.55″	8.33	15.96
	Yuexigong Jiao	ZB1	Qingsong Village Banqiao Town Yuexi County Sichuan Province China	102°33′59.94″	28°45′40.16″	7.50	20.37
	Yuexigong Jiao	ZB1	Qingsong Village Banqiao Town Yuexi County Sichuan Province China	102°33′59.22″	28°45′45.75″	6.24	14.43
	Yuexigong Jiao	ZB1	Qingsong Village Banqiao Town Yuexi County Sichuan Province China	102°33′58.22″	28°45′44.74″	6.66	20.43
	Yuexigong Jiao	ZB1	Hongguang Village Naituo Town Yuexi County Sichuan Province China	102°36′47.95″	28°42′38.35″	5.81	12.43
	Yuexigong Jiao	ZB1	Hongguang Village Naituo Town Yuexi County Sichuan Province China	102°36′47.8″	28°42′37.45″	3.33	12.99
	Yuexigong Jiao	ZB1	Hongguang Village Naituo Town Yuexi County Sichuan Province China	102°36′46.95″	28°42′35.43″	6.66	13.56
	Yuexigong Jiao	ZB1	Ebu Village Gu'er Town Yuexi County Sichuan Province China	102°41′18″	28°25′34″	5.42	14.81
	Yuexigong Jiao	ZB1	Ebu Village Gu'er Town Yuexi County Sichuan Province China	102°41′56.76″	28°25′15.32″	4.99	13.00
	Yuexigong Jiao	ZB1	Ebu Village Gu'er Town Yuexi County Sichuan Province China	102°41′26.89″	28°25′14.84″	5.00	14.61
	Lingshang Zhenglujiao	ZB2	Renyi Village Zeyuan Town Mianning County Sichuan Province China	102°4′53.202″	28°12′21.011″	5.42	11.28
	Lingshang Zhenglujiao	ZB2	Songlin Village Manshuiwan Town Mianning County Sichuan Province China	102°10′1.56″	28°12′17.48″	9.16	14.23
	Lingshang Zhenglujiao	ZB2	Luba Village Tuowu Town Mianning County Sichuan Province China	102°19′25.227″	28°47′48.82″	5.41	15.47
	Lingshang Zhenglujiao	ZB2	Huangjiaba Village Tuowu Town Mianning County Sichuan Province China	102°18′37.257″	28°47′48.82″	8.33	12.15
	Lingshang Zhenglujiao	ZB2	Dabaozi Village Caogu Town Mianning County Sichuan Province China	102°14′36″	28°38′21″	6.66	16.00
	Lingshang Zhenglujiao	ZB2	Cheyang Village Caogu Town Mianning County Sichuan Province China	102°15′31″	28°40′54″	7.08	11.56
	Lingshang Zhenglujiao	ZB2	Jinguang Village Jinping Town Mianning County Sichuan Province China	101°51′29.706″	28°27′19.162″	4.16	11.21
	Lingshang Zhenglujiao	ZB2	Jinguang Village Jinping Town Mianning County Sichuan Province China	101°51′1.866″	28°27′44.86″	7.50	16.24
	Lingshang Zhenglujiao	ZB2	Jiaoding Village Shaba Town Mianning County Sichuan Province China	102°2′54.348″	28°10′57.063″	7.91	13.62
	Lingshang Zhenglujiao	ZB2	Jiaoding Village Shaba Town Mianning County Sichuan Province China	102°2′18.37″	28°11′5.629″	7.50	18.55
	Lingshang Zhenglujiao	ZB2	Daqiao Village Daqiao Town Mianning County Sichuan Province China	102°13′49.437″	28°42′1.894″	3.33	11.03
	Lingshang Zhenglujiao	ZB2	Dayanjing Village Wuhai Mianning Sichuan Province China	102°15′38″	28°43′37″	3.75	16.77
	Lingshang Zhenglujiao	ZB2	Shigu Village Huilong Town Mianning County Sichuan Province China	102°07′32.1″	28°28′47.5″	7.49	13.26
	Lingshang Zhenglujiao	ZB2	Tianba Village Tianba Luding County Sichuan Province China	102°14′17.02″	29°54′2.358″	2.50	12.22
	Da Hongpao	ZB3	Shuitang Village Zhenping Town Songpan County Sichuan Province China	103°45′53.575″	32°13′9.126″	7.08	12.56
	Da Hongpao	ZB3	Shuitang Village Zhenping Town Songpan County Sichuan Province China	103°46′57′	32°13′14′	5.41	9.71
	Da Hongpao	ZB3	Suoqiao Village Yanmen Town Wenchuan County Sichuan Province China	103°37′41.669″	31°28′52.577″	4.58	12.85
	Da Hongpao	ZB3	Xinmin Village Zhenping Town Songpan County Sichuan Province China	103°52′5.77″	32°14′6.519″	5.42	10.40
	Da Hongpao	ZB3	Xinmin Village Zhenping Town Songpan County Sichuan Province China	103°52′32.539″	32°13′44.033″	5.41	10.47
	Da Hongpao	ZB3	Mayizu Town Jinyang County Sichuan Province China	103°17′1.154″	27°41′4.056″	6.25	15.98
	Da Hongpao	ZB3	Taiping Village Taiping Town Mao County Sichuan Province China	103°40′41.77″	32°1′17.501″	5.83	10.95
	Hanyuan Huajiao	ZB4	Xinli Village Qingxi Town Hanyuan County Sichuan Province China	102°35′12.155″	29°34′59.343″	3.33	13.05
	Hanyuan Huajiao	ZB4	Xinli Village Qingxi Town Hanyuan County Sichuan Province China	102°37′34″	29°36′02″	13.33	14.98
	Hanyuan Huajiao	ZB4	Shuangping Village Qingxi Town Hanyuan County Sichuan Province	102°36′44.682″	29°35′49.286″	6.66	9.94
	Hanyuan Huajiao	ZB4	Guanhua Village Yidong Town Hanyuan County Sichuan Province China	102°26′11.65″	29°41′10.727″	6.25	15.50
	Hanyuan Huajiao	ZB4	Guanhua Village Yidong Town Hanyuan County Sichuan Province China	102°26′13.792″	29°41′9.657″	7.50	18.23
	Hanyuan Huajiao	ZB4	Dadi Village Liyuan Town Hanyuan County Sichuan Province China	102°24′53″	29°35′19″	10.83	20.16
	Hanyuan Huajiao	ZB4	Gaoqiao Village Sanjiao Yidong Town Hanyuan County Sichuan Province China	102°24′37″	29°41′64″	10.42	14.23

**Figure 2:**
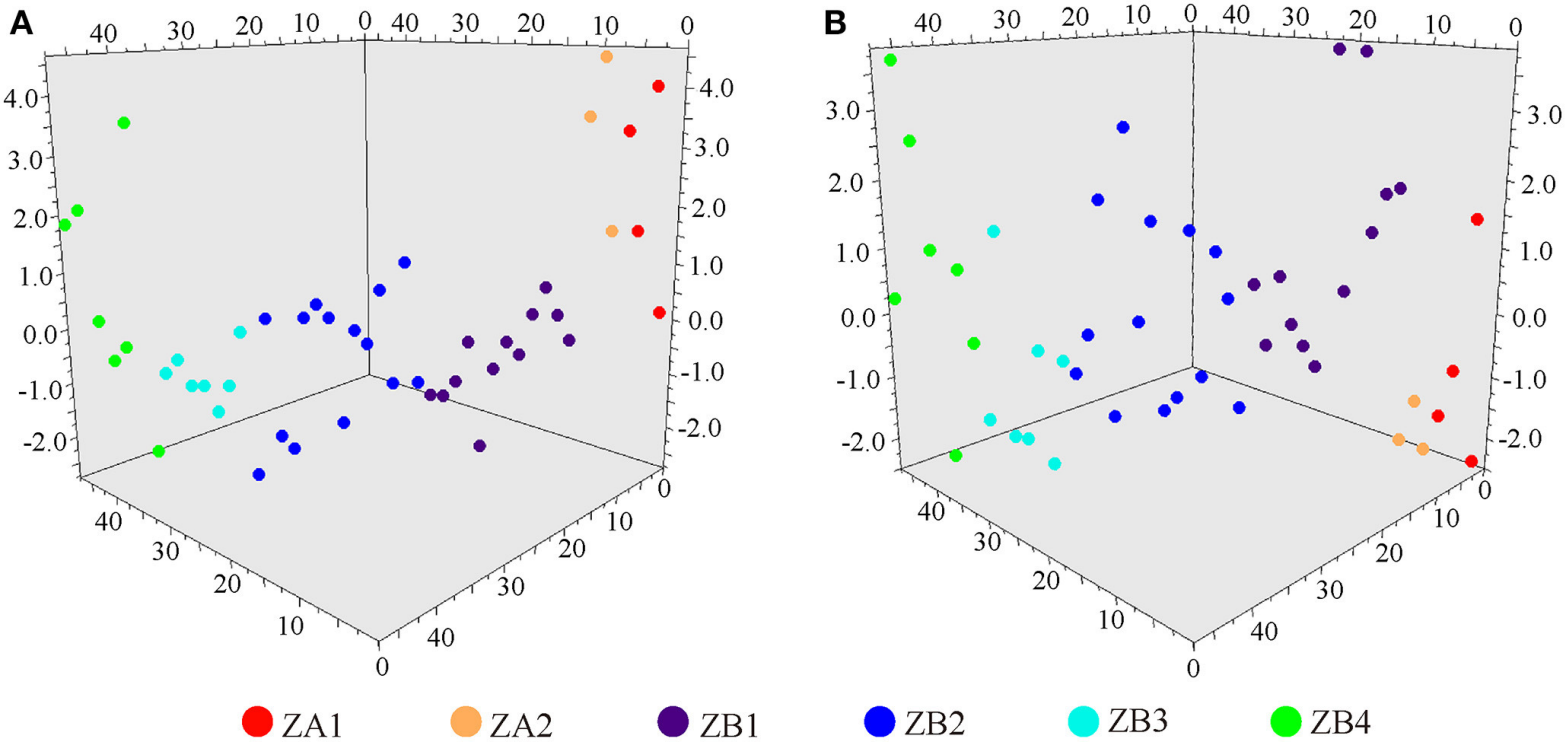
**PCA analysis based on the contents of volatile oil and non-volatile ether extract of six Szechuan pepper cultivars. (A)** PCA analysis for volatile oil content, **(B)** PCA analysis for non-volatile ether extract content, ZA1 Jiyang Qinghuajiao, ZA2 Tengjiao, ZB1 Yuexigong Jiao, ZB2 Linshan Zhenglujiao, ZB3 Da Hongpao, and ZB4 Hanyuan Huajiao.

A further PCA analysis indicated that the *Z. armatum* and *Z. bungeanum* cultivars could be clearly distinguished from each other based on the volatile oil and non-volatile ether extract contents, respectively. Two *Z. armatum* cultivars (ZA1 and ZA2) were located in the upper area of the PCA 3D scatter plot for volatile oil, and most of the *Z. bungeanum* cultivars (ZB1, ZB2, ZB3, and ZB4) were distributed in the lower part. However, *Z. bungeanum* cultivars were distributed in the upper quadrants of the PCA 3D scatter plot for non-volatile ether extract and two *Z. armatum* cultivars were located in the lower region (Figures 2A,B). These results matched the results for the volatile oil and non-volatile ether extract contents.

### Genetic diversity analysis

**Figure 3:**
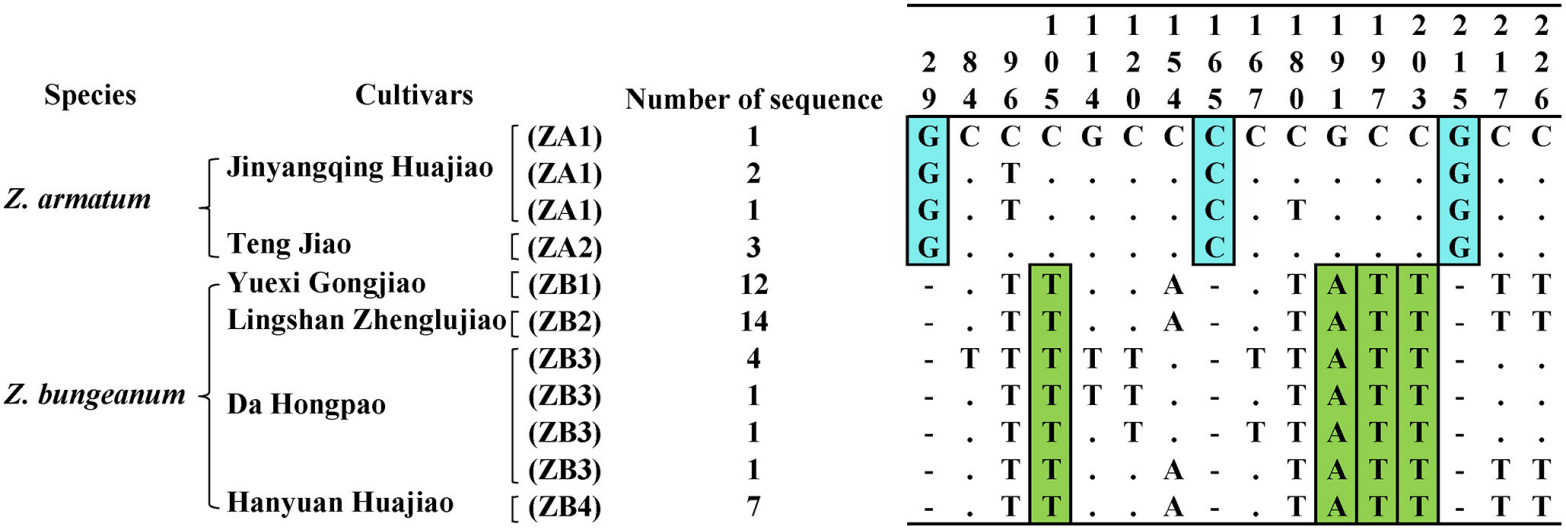
**Variable sites and insertions/deletions for six Szechuan pepper cultivars based on ITS2 sequences**. The specific variable sites and deletions are highlighted.

The success rates for both DNA extraction and PCR amplification were 100%. The sequencing results indicated that highly-qualified bidirectional trace files were located in the ITS2 regions. The ITS2 sequence lengths for six Szechuan pepper cultivars and their related species ranged from 222 to 227 bp, and the average GC content was 70.29% (Supplementary Table S3). The genetic distances for the ITS2 sequence based on a Kimura 2-Parameter (K2P) model indicated that the minimum interspecific distances for all Szechuan peppers and their related species were higher than those of the maximum intraspecific distances, except in the case of *Z. bungeanum* (Supplementary Table S4). According to the phylogenetic tree analysis, *Z. armatum* and *Z. bungeanum* can be distinguished from each other as well as from the other seven related species based on an ITS2 barcode (Supplementary Figure S1). However, the cultivars could not be distinguished because the cultivars of each species shared same haplotype (Figure 3). Moreover, the sequences characters indicated that single nucleotide polymorphisms (SNPs) provide insight into the species discrimination: the SNPs occurred only at the species level, not in the cultivars (Figure 3, Supplementary Figure S2). For *Z. armatum*, three unique inserts exited at site29, site165, and site 215, whereas ZA1 had high divergence. Nevertheless, *Z. bungeanum* had four unique variable sites that exited at site105, site 191, site 197 and site 203 and ZB3 had high divergence. The genetic distances of ZA1 and ZB3 ranged from 0~0.0089 and 0~0.0320, respectively (Supplementary Table S5). Therefore, the genetic variation of ZA1 and ZB3 was higher than other cultivars in each species, and no genetic variation occurred in ZA2, ZB1, ZB2, and ZB4. In other words, ZA2, ZB1, ZB2, and ZB4 were pure cultivars after long-term artificial processes.

### Influence of ecological factors on chemical composition and suitable habitat prediction

**Figure 4:**
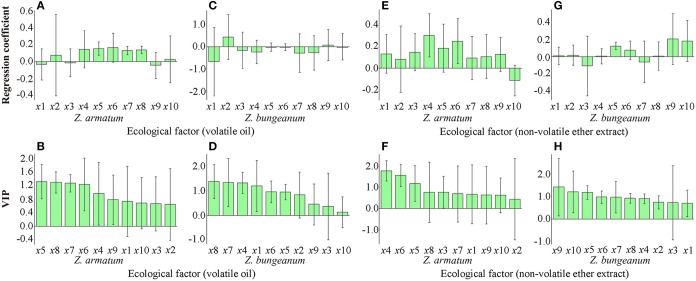
**Regression coefficient of ecological factors (A,C,E,G) and VIP (B,D,F,H) of volatile oil and non-volatile ether extract contents in *Z. armatum* and *Z. bungeanum* (the whiskers represent standard deviation)**. *x*1 Elevation, *x*2 Active accumulated temperature, *x*3 Sunshine duration, *x*4 Average annual temperature, *x*5 Minimum temperature in January, *x*6 Average temperature in January, *x*7 Maximum temperature in July, *x*8 Average temperature in July, *x*9 Annual precipitation, *x*10 Relative humidity. VIP scores larger than 1 indicated the most significant ecological factors, and the scores between 1 and 0.5 indicated the significant ecological factors, and the scores lower than 0.5 indicated the unimportant ecological factors.

According to the PLSR analysis, all of the ecological factors were positively correlated with the volatile oil contents of *Z. armatum*, except for the elevation and sunshine duration and the annual precipitation (Figure 4A). VIP scores of the minimum temperature in January (1.32), average temperature in July (1.29), maximum temperature in July (1.27), average temperature in January (1.24) were larger than 1 indicated most significant ecological factors influencing the contents of the *Z. armatum* volatile oil (Figure 4B). Only the relative humidity exhibited a negative correlation with the non-volatile ether extract contents of *Z. armatum* (Figure 4E), but the mean annual temperature (1.80), average temperature in January (1.56), and minimum temperature in January (1.17) were most significant ecological factors and the active accumulated temperature (0.43) is an unimportant ecological factor (Figure 4F).

Two ecological factors, active accumulated temperature and annual precipitation, demonstrated a positive correlation with the volatile oil content of *Z. bungeanum* (Figure 4C), however, the average temperature in July (1.40), maximum temperature in July (1.36), mean annual temperature (1.34), and elevation (1.21) were extremely significant ecological factors (Figure 4D), and the annual precipitation (0.46), sunshine duration (0.38) relative humidity (0.13) were unimportant ecological factors. Furthermore, the sunshine duration and maximum temperature in July had a negative influence on the non-volatile ether extract contents of *Z. bungeanum* (Figure 4G), but the annual precipitation (1.42), relative humidity (1.21) and minimum temperature in January (1.18) were most important ecological factors (Figure 4H).

**Figure 5:**
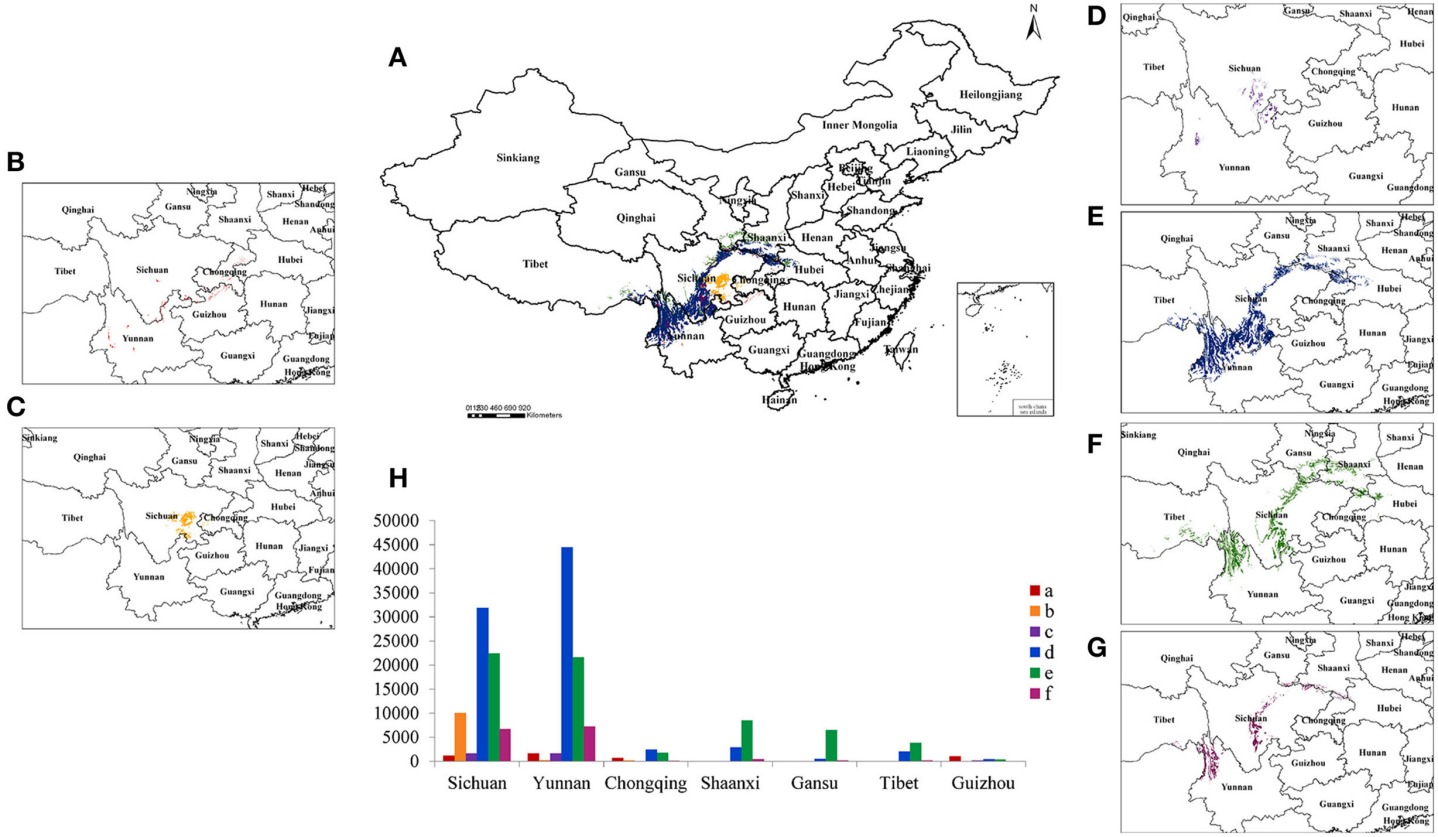
**Regional division of suitable production areas for Szechuan pepper cultivars in China were predicted by a self-developed and GIS-based computer program, RISTCM. (A)** Regional division of suitable production areas of Szechuan pepper cultivars in China (SI 95~100%), **(B)** Jinyangqing Huajiao, **(C)** Tengjiao, **(D)** Yuexigong Jiao, **(E)** Linshang Zhenglujiao, **(F)** Da Hongpao, **(G)** Hanyuan Huajiao, **(H)** and suitable provinces for Szechuan pepper cultivars with a similarity index of 95~100%.

Based on these 10 vital ecological factors and soil types, the suitable habitat for each cultivar was predicted by RISTCM software (Figure 5). The suitable production areas for *Z. armatum* and *Z. bungeanum* rarely overlap, but the cultivars belonging to *Z. bungeanum* did overlap. The cultivars belonging to *Z. bungeanum* can theoretically survive across much of the Hengduan Mountains, Ta-pa Mountains and Qinling Mountains. ZA2, belonging to *Z. armatum*, was mainly distributed in the Sichuan Basin, and ZA1 was mainly distributed in the Dalou Mountains and Wu Mountains.

RISTCM prediction indicated that 9 provinces (including 293 cities and counties) were suitable for growing six Szechuan pepper cultivars. Furthermore, the area was as large as approximately 185, 506 km^2^ (SI 95~100%), and Sichuan Province was shown to be the largest suitable area for cultivating all six cultivars (Supplementary Table S6). For the *Z. armatum* cultivars, the Sichuan Basin and Dalou Mountains were predicted to be the primary suitable cultivation region. However, along the Ta-pa Mountains, Qinling Mountains and Hengduan Mountains, *Z. bungeanum* was more suitable for cultivation.

## Discussion

As important edible, medicinal, and economic plants, Szechuan peppers and their related plants are central to traditional Chinese cultures and face an increase in demand along with improvement of quality control. High-quality raw materials from Szechuan peppers that have adequate nutritional value, desirable taste and food safety are fundamental to food production (Konczak and Roulle, 2011).

In previous reports, multiple volatile oil, amides and alkaloids have been demonstrated to be the most bioactive and odorous compounds of Szechuan peppers (Wang et al., 2011; Brijwal et al., 2013). Among those compounds, volatile oil is the principle source of the special flavor in Szechuan peppers and the main indicator of aroma intensity (Singh et al., 2013). Another compound that creates numbness in the mouth originates from unsaturated fatty acid amides, which are defined as a non-volatile ether extract (Jang et al., 2008). The numb quality is strictly described as a tingling paresthesia and numbing sensation. Therefore, these two chemical compositions are the economically valuable indices for Szechuan pepper production. The Commerce Department, General Administration of Quality Supervision, Inspection and Quarantine and Standardization Administration of China also regulated the two chemical compositions as the evaluation indices for quality control (Commerce Department Standard of the People's Republic of China, 1992; General Administration of Quality Supervision, Inspection and Quarantine of the People's Republic of China, Standardization Administration of the People's Republic of China, 2008). According to those two national standards, the lowest volatile oil content and non-volatile ether extract content in first-class Szechuan peppers are established at 2.5 and 2.0%, respectively.

By analyzing the chemical compounds of 47 samples, we concluded that *Z. armatum* contains more volatile oil, and *Z. bungeanum* contains more non-volatile ether extract. Furthermore, ZA2 contains the most volatile oil and ZB1 contains the most non-volatile ether extract. Moreover, all of the investigated samples achieved the first-class standard or higher. ZA1 showed relatively higher content of both volatile oil and non-volatile ether extract among the six cultivars. Species and cultivars exhibited huge disparity in their content of volatile oil and non-volatile ether extract according to a one-way ANOVA.

In recent years, studies have proven that the combined chemical and molecular method generally and accurately identified and classified species, subspecies and cultivars and efficiently controlled and evaluated the quality of medicinal plants (Djabou et al., 2011; Dong et al., 2012; Ali et al., 2013; Zheng et al., 2014), crops (Caligiani et al., 2014), and food (Snyder and Worobo, 2014). In this paper, a chemical and molecular analysis was jointly used to construct a clear classification of the different types Szechuan peppers. According to the data of sequence characteristics, intra- and interspecific K2P genetic distances and an NJ algorithm, relatively large variation occurred only at the species level but not at the cultivar level. The low level of variation of nuclear ribosomal ITS2 sequences within Szechuan pepper cultivars may stem from the domestication process and the low turnover caused by the long life span of plants. The sequence characteristics also revealed that ZA1 and ZB3 exhibit more divergence than other cultivars, probably resulting from wildly distributed areas with diverse environments. The long-term cultivation environment might be responsible for small genetic changes within cultivars. However, a chemical analysis indicated differences between species and cultivars. On the basis of volatile oil and non-volatile ether extract contents, the PCA results determined that the six cultivars were classified into their own groups, especially between the two focal species. Overall, Szechuan peppers exhibited detectable levels of variation in their chemical components and ITS2 sequences, which enabled the producers to protect and certify products, and provide quality inspection institutions with the ability to spot adulterants and identify fraudulent samples; this also allowed consumers to confidently purchase quality Szechuan peppers.

The PLSR analysis thoroughly illustrated the extent of influence of ecological factors on each chemical composition. For example, *Z. bungeanum* was dominated by the non-volatile ether extract; therefore, it created a stronger numbing sensation in the mouth. As disclosed in the PLSR results, the annual precipitation, temperature in January and relevant humidity have very significant positive correlation with the non-volatile ether extract contents of *Z. bungeanum*. *Z. bungeanum* has been reported to favor humid air and soil (Zhang et al., 2011; Sun et al., 2014). Therefore, the suitable production areas of ZB1, ZB2, ZB3, and ZB4, predicted by RISTCM, were along the Ta-pa Mountains, Qinling Mountains and Hengduan Mountains, in favorably subtropical environments having an average annual temperature of 17.1°C and 800~1400 mm of annual precipitation. The climate features in these areas are perfectly suited to growing *Z. bungeanum* cultivars.

In contrast to *Z. bungeanum*, the volatile oil and non-volatile ether extract concentrations in *Z. armatum* were significant positively correlated with temperature, including the mean annual temperature, minimum and average temperature in January, and maximum and average temperature in July. However, less annual precipitation and low elevation contributed to higher volatile oil content. Therefore, the suitable habitats for *Z. armatum* cultivars were mainly spread across the southeastern Sichuan Basin. The warm and arid climate of the Chongqing, Yunnan, and Sichuan provinces, with the mean annual temperature and annual precipitation reaching 20.5°C and 967 mm, respectively, stimulated the accumulation of volatile oil and resulted in a stronger unique flavor and less numbing sensation (Zhang et al., 2011). Furthermore, the fact that ZA1 contains more volatile oil and non-volatile ether extract may be attributed to its special distribution areas, including the Dalou Mountains and Wu Mountains (Figure 5D). The average annual temperature in its distribution areas is higher than ZA2. This factor supports the volatile oil and non-volatile ether extract accumulation.

In general, appropriate precipitation and temperature are the key factors supporting the accumulation of flavor compounds. Therefore, the difference between the quantity of two important chemical compositions separately in *Z. armatum* and *Z. bungeanum* arose from different ecologic systems. The adaptation of these species incorporated all ecological factors that are good for synthesis and the growth of chemical components, which may be the reasons why suitable cultivation regions for these cultivars in *Z. bungeanum* overlapped.

In this study, we have proven that the contents of volatile oil and non-volatile ether extract and ITS2 sequence were different in *Z. armatum* and *Z. bungeanum*. The ability of a combination chemical and molecular method to classify cultivars enables a supervisory body to better guarantee food authentication and safety. The suitable production areas that were predicted based on ecological similarity between sample sites could be used as a preliminary selection process for Szechuan peppers cultivar production. Based on these results, further studies are needed for a more reliable and representative quality assessment of Szechuan pepper cultivars.

## Author contributions

LX, SC, and MY conceived the study and participated in its design. LX, YL, and XL analyzed the data and drafted the manuscript. YY and CX conducted the experiments. All authors have read and approved the final manuscript.

### Conflict of interest statement

The authors declare that the research was conducted in the absence of any commercial or financial relationships that could be construed as a potential conflict of interest.
