# Supplementary material for: The Chemical and Genetic Characteristics of Szechuan Pepper (Zanthoxylum bungeanum and Z. armatum) Cultivars and Their Suitable Habitat
Source: Front Plant Sci. 2016 Apr 19;7:467. doi: 10.3389/fpls.2016.00467 (PMC4835500; doi:10.3389/fpls.2016.00467)
Supplement: Supplementary file 1 [file Table1.PDF]

Supplementary Material

The chemical and genetic characteristics of Szechuan pepper cultivars and their suitable habitat

Li Xiang[1], Yue Liu[1] Caixiang Xie [2], Xiwen Li[1], Yadong Yu[1, 3], Meng Ye[3 *], Shilin Chen[1 *]

***Correspondence:**

Shilin Chen

slchen@icmm.ac.cn

Meng Ye

yemeng5581@163.com

Supplementary Table 1 The information of Szechuan pepper cultivars characters and cultivation points

Species	Cultivar	Cultivar No.	Tree age	Harvest time	Harvest stage	Characters	Cultivation points
Z. armatum	Jinyangqing Huajiao (ZA	CHUANS-SV-ZA-002-2013	8-10years	2014. 07	Ripe stage	Bright green pericarp and contains more special aromatic smell. Fruiting early, high and stable yield, resistance to drought , disease and insect, etc.	The thickness of soil layer is above 50cm; well-draining high-calcium soil and purple sandstone weathering soil with evaluation of 800～1800 m are suitable for the cultivation.
	Tengjiao (ZA2)	CHUANS-SV-ZA-001-2014	8-10years	2014. 07	Ripe stage	Bright green pericarp. Reletive larger-diameter fruits with special aromatic smell. Seeding produce fruit in the next year.	Sandy soil, purple soil or yellow soil with pH 5.5～7.5 in Sichuan Basin at evaluation below 1200 m are suitable for the cultivation. Shady slope and paddy fields are not suitable for the cultivation.
Z. bungeanum	Yuexigong Jiao (ZB1)	CHUANR-SV-ZB-019-2011	8-10years	2014. 08	Ripe stage	Bright red pericarp in mature period and dark red after drying. Strong verrucosa glandular opaque spots and more numbing sensation.	Well-draining loose soil in sunny or hail-sunny slope at evaluation from 1000 to 2600 m are suitable for the cultivation.
	Linshang Zhenglujiao (Z	CHUANR-SV-ZB-018-2011	8-10years	2014. 08	Ripe stage	Bright red pericarp and the fruits diameter can reach 4.8 mm.Strong numbing sensation. Resistance to drought, barren and cold.	Well-draining loose soil in sunny or hail-sunny slope at evaluation from 1000 to 2600 m are suitable for the cultivation.
	Da Hongpao (ZB3)	CHUANR-SV-ZB-008-2014	8-10years	2014. 08	Ripe stage	Bright red pericarp. Shallow-rooted plant with developed lateral root. strong numbing sensation.	Dry climate, the soil pH value of 6 ~ 8, and evaluation below 2700 m are suitbale for the ZB3 cultivation.
	Hanyuan Huajiao (ZB4)	CHUANS-SV-ZB-003-2012	8-10years	2014. 08	Ripe stage	Bright red pericarp. Reletive larger-diameter fruits and with thicker pericarp. Three small gain growing with fruits. Strong numbing sensation. Resistance to drought, barren and cold.	Sandy soil, purple soil or yellow soil with pH 4.5～8.0 with wide evaluation from 1000 to 2800 m are suitable for the cultivation.
