# Supplementary material for: The Chemical and Genetic Characteristics of Szechuan Pepper (Zanthoxylum bungeanum and Z. armatum) Cultivars and Their Suitable Habitat
Source: Front Plant Sci. 2016 Apr 19;7:467. doi: 10.3389/fpls.2016.00467 (PMC4835500; doi:10.3389/fpls.2016.00467)
Supplement: Supplementary file 2 [file Table2.PDF]

Supplementary Material

The chemical and genetic characteristics of Szechuan pepper cultivars and their suitable habitat

Li Xiang[1], Yue Liu[1] Caixiang Xie [2], Xiwen Li[1], Yadong Yu[1, 3], Meng Ye[3 *], Shilin Chen[1 *]

***Correspondence:**

Shilin Chen

slchen@icmm.ac.cn

Meng Ye

yemeng5581@163.com

Supplementary Table 2 Different species and cultivars of Szechuan peppers for DNA barcodes

Latin Name	Voucher No.	Haplotype	Locality	GenBank No.
Z. armatum	YC0730MT09	A1	Ribu Village Taoping Town Jinyang County Sichuan Povince China	KP642516
	YC0730MT10	A2	Luxiang Village Honglian Town Jinyang County Sichuan Povince China	KP642517
	YC0730MT38	A1	Zhige Town Hongya County Sichuan Province China	KP642518
	YC0730MT21	A1	Wulong Village Zhige Town Hongya County Sichuan Province China	KP642520
	YC0730MT86	A2	Qingsong Village Jinyang County Sichuan Povince China	KP642521
	YC0730MT32	A1	Doudan Village Beijiao Town Ya'an County Sichuan Province China	KP642522
	YC0730MT34	A1	Hongya County Sichuan Province China	KP642523
	YC0730MT36	A2	Hongya County Sichuan Province China	KP642524
	YC0730MT16	A1	Hongya County Sichuan Province China	KP642526
	YC0730MT13	A3	Mianning County Sichuan Province China	KP642527
	YC0730MT30	A1	Xishuangbanna City Yunnan Province China	KP642528
	YC0730MT31	A1	Xishuangbanna City Yunnan Province China	KP642529
	YC0730MT18	A1	Chongqing China	KP642530
	YC0730MT12	A4	Kangding City Ganzi State Sichuan Province China	KP642531
	YC0730MT29	A2	Wanzhou City Chongqing China	KP642532
	YC0730MT11	A5	Guangming Village Pailai Town Jinyang County Sichuan Povince China	KP642533
	YC0730MT07	A2	Hanyuan County Ya'an City Sichuan Povince China	KP642534
	YC0730MT08	A2	Hanyuan County Ya'an City Sichuan Povince China	KP642535
	YC0730MT40	A1	Nanchuan County Chongqing China	KP642536
	YC0730MT41	A3	Nanchuan County Chongqing China	KP642537
	YC0730MT42	A3	Nanchuan County Chongqing China	KP642538
	YC0730MT28	A3	Beijing Chinese Academy of Medical Sciences Beijing China	KP642539
	YC0730MT27	A3	Beijing Chinese Academy of Medical Sciences Beijing China	KP642540
	YC0730MT22	A6	Foping County Hanzhong City Shanxi Province China	KP642541
	YC0730MT23	A6	Foping County Hanzhong City Shanxi Province China	KP642542
	YC0730MT39	A1	Beijing Chinese Academy of Medical Sciences Beijng China	KP642543
	YC0730MT05	A3	Anhui University of Chinese Medicine Anhui Province China	KP642544
	YC0730MT06	A3	Anhui University of Chinese Medicine Anhui Province China	KP642545
	YC0730MT03	A3	Beijing Chinese Academy of Medical Sciences Beijing China	KP642546
	YC0730MT04	A7	Beijing Chinese Academy of Medical Sciences Beijing China	KP642547
Z. bungeanum	YC0167MT08	B1	Qingsong Village Banqiao Town Yuexi County Sichuan Provine China	KP642548
	YC0167MT09	B1	Qingsong Village Banqiao Town Yuexi County Sichuan Provine China	KP642549
	YC0167MT40	B1	Qingsong Village Banqiao Town Yuexi County Sichuan Provine China	KP642550
	YC0167MT10	B1	Qingsong Village Banqiao Town Yuexi County Sichuan Provine China	KP642551
	YC0167MT41	B1	Qingsong Village Banqiao Town Yuexi County Sichuan Provine China	KP642552
	YC0167MT11	B1	Qingsong Village Banqiao Town Yuexi County Sichuan Provine China	KP642553
	YC0167MT12	B1	Qingsong Village Banqiao Town Yuexi County Sichuan Provine China	KP642554
	YC0167MT13	B1	Qingsong Village Banqiao Town Yuexi County Sichuan Provine China	KP642555
	YC0167MT14	B1	Qingsong Village Banqiao Town Yuexi County Sichuan Provine China	KP642556
	YC0167MT15	B1	Ebu Village Gu'er Town Yuexi County Sichuan Province China	KP642557
	YC0167MT16	B1	Ebu Village Gu'er Town Yuexi County Sichuan Province China	KP642558
	YC0167MT17	B1	Ebu Village Gu'er Town Yuexi County Sichuan Province China	KP642559
	YC0167MT22	B1	Renyi Village Zeyuan Town Mianning County Sichuan Province China	KP642560
	YC0167MT23	B1	Songlin Village Manshuiwan Town Mianning County Sichuan Province China	KP642561
	YC0167MT24	B1	Luba Village Tuowu Town Mianning County Sichuan Province China	KP642562
	YC0167MT25	B1	Huangjiaba Village Tuowu Town Mianning County Sichuan Province China	KP642563
	YC0167MT26	B1	Caogudabaozi Village Mianning County Sichuan Province China	KP642564
	YC0167MT27	B1	Cheyang Village Caogu Town Mianning County Sichuan Province China	KP642565
	YC0167MT46	B1	Jinguang Village Jinping Towm Mianning County Sichuan Province China	KP642566
	YC0167MT29	B1	Jinguang Village Jinping Towm Mianning County Sichuan Province China	KP642567
	YC0167MT56	B1	Jiaoding Village Shaba Town Mianning County Sichuan Province China	KP642568
	YC0167MT30	B1	Jiaoding Village Shaba Town Mianning County Sichuan Province China	KP642569
	YC0167MT62	B1	Shuitang Village Zhenping Town Songpan County Sichuan Province China	KP642570
	YC0167MT32	B1	Luba Village Tuowu Town Mianning County Sichuan Province China	KP642571
	YC0167MT33	B1	Daqiao Village Daqiao Town Mianning County Sichuan Province China	KP642572
	YC0167MT34	B1	Dayanjing Village Wuhai Mianning Sichuan Province China	KP642573
	YC0167MT43	B1	Shigulu Village Huilong Town Mianning City Sichuan Province China	KP642574
	YC0167MT44	B1	Tianba Village Tianba Town Luding County Sichuan Province China	KP642575
	YC0167MT49	B1	Daqiao Village Daqiao Town Mianning County Sichuan Province China	KP642576
	YC0167MT35	B2	Shuitang Village Zhenping Town Songpan County Sichuan Province China	KP642577
	YC0167MT36	B3	Shuitang Village Zhenping Town Songpan County Sichuan Province China	KP642578
	YC0167MT37	B4	Suoqiao Village Yanmen Town Wenchuan County Sichuan Province China	KP642579
	YC0167MT38	B2	Xinmin Village Zhenping Town Songpan County Sichuan Province China	KP642580
	YC0167MT39	B2	Xinmin Village Zhenping Town Songpan County Sichuan Province China	KP642581

	YC0167MT58	B1	Mayizu Town Jinyang County Sichuan Province China	KP642582
	YC0167MT59	B1	Xinli Village Qingxi Town Hanyuan County Sichuan Province China	KP642583
	YC0167MT06	B1	Xinli Village Qingxi Town Hanyuan County Sichuan Province China	KP642584
	YC0167MT20	B1	Shuangping Village Qingxi Town Hanyuan County Sichuan Province China	KP642585
	YC0167MT19	B1	Guanhua Village Yidong Town Hanyuan County Sichuan Province China	KP642586
	YC0167MT60	B1	Guanhua Village Yidong Town Hanyuan County Sichuan Province China	KP642587
	YC0167MT61	B1	Dadi Village Liyuan Town Hanyuan County Sichuan Province China	KP642588
	YC0167MT63	B5	Zhenping Village Zhenping Town Songpan County Sichuan Province China	KP642589
	YC0167MT83	B2	Yunxi County Shiyan City Hubei Province China	KP642590
	YC0167MT84	B1	Suining City Sichuan Province China	KP642591
	YC0167MT07	B1	Hongya Sichuan Province China	KP642592
	YC0167MT05	B1	Hanyuan Sichuan Province China	KP642593
	YC0167MT21	B2	Taiping Village Taiping Town Maoxian County Sichuan Province China	KP642594
	YC0167MT04	B1	Sanjiao Village Yidong Town Hanyuan County Sichuan Province China	KP642595
	YC0167MT64	B1	Hongya County Sichuan Province China	KP642596
	YC0167MT65	B6	Linxia City Gansu Province China	KP642597
	YC0167MT66	B7	Tianshui City Gansu Province China	KP642598
	YC0167MT67	B1	Shangluo County Shanxi Province China	KP642599
	YC0167MT68	B1	Shangluo County Shanxi Province China	KP642600
	YC0167MT69	B1	Fuping County Shanxi Province China	KP642601
	YC0167MT70	B1	Fuping County Shanxi Province China	KP642602
	YC0167MT71	B2	Lixian County Gansu Province China	KP642603
	YC0167MT77	B8	Hanyuan County Sichuan Province China	KP642604
	YC0167MT80	B9	Nanchuan County Chongqing China	KP642605
	YC0167MT82	B1	Pingheliang Town Ningshan County Shanxi Province China	KP642606
Z. nitidum	YC0458MT28	E1	Huanghuapo Village Pingyuan County Guangdong Province China	KP642614
	YC0458MT04	E2	Yongning County Nanning City Guangxi Province China	KP642615
	YC0458MT02	E3	Datang County Nanning City Guangxi Province China	KP642616
	YC0458MT05	E4	Chongdong Town Xinxing County Guangdong Province China	KP642617
Z. dissitum	YC0733MT03	C1	Beijing Chinese Academy of Medical Sciences Beijing China	KP642607
	YC0733MT02	C2	Botanical Garden Xi’an Shaanxi Province China China	KP642608
	YC0733MT01	C2	Botanical Garden Xi’an Shaanxi Province China China	KP642609
Z. myriacanthum	YC0734MT01	D1	Xishuangbanna CityYunnan Province China	KP642610
	YC0734MT04	D1	Xishuangbanna CityYunnan Province China	KP642611
	YC0734MT03	D1	Xishuangbanna CityYunnan Province China	KP642612
	YC0734MT02	D1	Xishuangbanna CityYunnan Province China	KP642613
Z. ovalifolium var. *spinifolium*	YC0736MT01	E1	Shimen Reservoir Hanzhong City Shanxi Province China	KP642636
		E1	GenBank	HM851479
Z. ovalifolium	YC0735MT01	E2	Shimen Reservoir Hanzhong City Shanxi Province China	KP642618
	YC0735MT02	E1	Shimen Reservoir Hanzhong City Shanxi Province China	KP642619
Z. piasezkii	YC0737MT13	F1	Shimen Reservoir Hanzhong City Shanxi Province China	KP642620
	YC0737MT02	F1	Botanical Garden Xi’an Shaanxi Province China	KP642621
Z. schinifolium	YC0731MT04	G1	Liaoning Province China	KP642624
	YC0731MT05	G1	Liaoning Province China	KP642625
	YC0731MT06	G1	Liaoning Province China	KP642626
	YC0731MT07	G1	Baoding City Hebei Province China	KP642627
	YC0731MT08	G1	Baoding City Hebei Province China	KP642628
	YC0731MT09	G1	Baoding City Hebei Province China	KP642629
	YC0731MT10	G1	Baoding City Hebei Province China	KP642630
	YC0731MT11	G1	Baoding City Hebei Province China	KP642631
	YC0731MT12	G1	Baoding City Hebei Province China	KP642632
	YC0731MT01	G1	Shandong Province China	KP642633
	YC0731MT02	G1	Shandong Province China	KP642634
	YC0731MT03	G1	Shandong Province China	KP642635
Z. piperitum	YC1500MT02	H1	Toyama Japan	KP642622
	YC1500MT01	H1	Toyama Japan	KP642623
T. asiatica			GenBank	JF978976
			GenBank	JF978977
			GenBank	JF978978
