# Supplementary material for: The Chemical and Genetic Characteristics of Szechuan Pepper (Zanthoxylum bungeanum and Z. armatum) Cultivars and Their Suitable Habitat
Source: Front Plant Sci. 2016 Apr 19;7:467. doi: 10.3389/fpls.2016.00467 (PMC4835500; doi:10.3389/fpls.2016.00467)
Supplement: Supplementary file 3 [file Table3.PDF]

Supplementary Material

The chemical and genetic characteristics of Szechuan pepper cultivars and their suitable habitat

Li Xiang[1], Yue Liu[1] Caixiang Xie [2], Xiwen Li[1], Yadong Yu[1, 3], Meng Ye[3 *], Shilin Chen[1 *]

***Correspondence:**

Shilin Chen

slchen@icmm.ac.cn

Meng Ye

yemeng5581@163.com

Supplementary Table 3 The ecological factors of different Szechuan peppers cultivars

Species	Cultivars	Abbreviation	Locality	Evaluation/m	Active accumulated temperature/℃	Sunshine duration/h	Average annual temperature/℃	January		July		Annual precipitation/mm	Relative humidity/%	Soil type
								Minimum temperature/℃	Average temperature/℃	Maximum temperature/℃	Average temperature/℃			
Z. armatum	Jinyangqing Huajiao	ZA1	Ribu Village(G RBZZ) Taoping Town Jinyang County Sichuan Province China	1800	4165	1867	19.3	1.1	4.1	25.9	21.5	1068	74.4	cinnamon-red soil
	Jinyangqing Huajiao	ZA1	Luxiang Village(G WCP) Honglian Town Jinyang County Sichuan Province China	1600	4819	1858	22.5	4.9	7.2	29.6	25.4	1025	75.2	limestone soil
	Jinyangqing Huajiao	ZA1	Guangming Village Pailai Town Jinyang County Sichuan Province China	1400	4981	1910	20.7	2.6	5.5	27.4	23.0	1051	74.3	limestone soil
	Jinyangqing Huajiao	ZA1	Qingsong Village (G SB) Jinyang County Sichuan Province China	1600	4902	1977	20.7	2.6	5.6	27.4	23.1	1050	74.6	limestone soil
	Tengjiao	ZA2	Zhige Town Hongya County Sichuan Province China	650	5228	1035	19.8	3.6	5.4	29.3	24.9	867	81.2	purple soil
	Tengjiao	ZA2	WulongVillage Zhige Town Hongya County Sichuan Province China	650	5177	1032	19.7	3.4	5.2	29.1	24.7	868	81.2	purple soil
	Tengjiao	ZA2	Doudan Village Beijiao Town Ya’an City Sichuan Province China	670	4369	989	20.5	4.2	6.1	29.9	25.5	844	79.0	purple soil
Z. bungeanum	Yuexigong Jiao	ZB1	Qingsong Village Banqiao Town Yuexi County Sichuan Province China	1950	2680	1711	17.0	-1.8	2.6	23.4	18.7	984	73.7	yellow brown soil
	Yuexigong Jiao	ZB1	Qingsong Village Banqiao Town Yuexi County Sichuan Province China	1950	2680	1711	17.0	-1.8	2.6	23.4	18.7	984	73.7	yellow brown soil
	Yuexigong Jiao	ZB1	Qingsong Village Banqiao Town Yuexi County Sichuan Province China	1970	2680	1711	17.0	-1.8	2.6	23.4	18.7	984	73.7	yellow brown soil
	Yuexigong Jiao	ZB1	Qingsong Village Banqiao Town Yuexi County Sichuan Province China	2145	2674	1711	17.1	-1.7	2.6	23.5	18.8	982	73.1	yellow brown soil
	Yuexigong Jiao	ZB1	Qingsong Village Banqiao Town Yuexi County Sichuan Province China	2130	2674	1711	17.1	-1.7	2.6	23.5	18.8	982	73.1	yellow brown soil
	Yuexigong Jiao	ZB1	Qingsong Village Banqiao Town Yuexi County Sichuan Province China	2125	2674	1711	17.1	-1.7	2.6	23.5	18.8	982	73.1	yellow brown soil
	Yuexigong Jiao	ZB1	Hongguang Village Naituo Town Yuexi County Sichuan Province China	1950	2306	1673	17.7	-0.8	3.2	24.3	19.7	980	73.6	purple soil
	Yuexigong Jiao	ZB1	Hongguang Village Naituo Town Yuexi County Sichuan Province China	1900	2306	1673	17.7	-0.8	3.2	24.3	19.7	980	73.6	purple soil
	Yuexigong Jiao	ZB1	Hongguang Village Naituo Town Yuexi County Sichuan Province China	1890	2306	1673	17.7	-0.8	3.2	24.3	19.7	980	73.6	purple soil
	Yuexigong Jiao	ZB1	Ebu Village Gu’er Town Yuexi County Sichuan Province China	2320	2309	1854	17.0	-2.1	2.2	23.1	18.3	1018	72.9	purple soil
	Yuexigong Jiao	ZB1	Ebu Village Gu’er Town Yuexi County Sichuan Province China	2301	2282	1854	16.8	-2.3	2.1	22.9	18.1	1022	73.0	purple soil
	Yuexigong Jiao	ZB1	Ebu Village Gu’er Town Yuexi County Sichuan Province China	2301	2309	1854	17.1	-1.9	2.3	23.2	18.5	1017	73.0	purple soil
	Linshang Zhenglujiao	ZB2	Renyi Village (G 1) Zeyuan Town Mianning County Sichuan Province China	1976	3990	2112	20.7	1.4	6.6	26.2	21.5	974	66.7	yellow brown soil
	Linshang Zhenglujiao	ZB2	Songlin Village (G 1) Manshuiwan Town Mianning County Sichuan Province China	630	3975	2106	20.6	1.5	6.5	26.2	21.5	977	67.2	yellow soil
	Linshang Zhenglujiao	ZB2	Luba Village (G 3) Tuowu Town Mianning County Sichuan Province China	2420	2138	1918	15.7	-3.9	1.4	21.3	16.6	991	70.5	yellow soil
	Linshang Zhenglujiao	ZB2	Huangjiaba Village (G 1) Tuowu Town Mianning County Sichuan Province China	2380	2125	1963	14.8	-5.1	0.6	20.2	15.4	1002	70.2	yellow soil
	Linshang Zhenglujiao	ZB2	Caogudabaozi Village (G 1) Mianning County Sichuan Province China	1990	3475	1775	18.2	-0.9	3.9	24.0	19.5	969	71.9	yellow soil
	Linshang Zhenglujiao	ZB2	Cheyang Village (G 8) Caogu Town Mianning County Sichuan Province China	2179	2214	1830	17.1	-2.2	2.9	22.8	18.2	980	71.3	yellow soil
	Linshang Zhenglujiao	ZB2	Jinguang Village (G SHHP) Jinping Towm Mianning County Sichuan Province China	2100	3530	1858	19.5	-0.3	5.3	24.9	20.2	956	67.7	yellow brown soil
	Linshang Zhenglujiao	ZB2	Jinguang Village (G SHHP) Jinping Towm Mianning County Sichuan Province China	2200	3490	1964	18.7	-1.4	4.5	23.9	19.2	966	67.2	yellow brown soil
	Linshang Zhenglujiao	ZB2	Jiaoding Village Shaba Town Mianning County Sichuan Province China	1900	4011	2143	19.1	-0.7	5.1	24.1	19.3	995	65.9	yellow brown soil
	Linshang Zhenglujiao	ZB2	Jiaoding Village Shaba Town Mianning County Sichuan Province China	1900	3935	2158	19.2	-0.4	5.3	24.2	19.6	992	65.9	yellow brown soil
	Linshang Zhenglujiao	ZB2	Daqiao Village Daqiao Town Mianning County Sichuan Province China	2102	3063	1941	16.8	-2.6	2.6	22.4	17.8	981	70.1	yellow soil
	Linshang Zhenglujiao	ZB2	Dayanjing Village (G 1) Wuhai Mianning Sichuan Province China	2090	2196	1777	17.6	-1.5	3.4	23.5	18.9	969	71.8	yellow soil
	Linshang Zhenglujiao	ZB2	Shigulu Village Huilong Town Mianning County Sichuan Province China	1906	3521	1865	18.6	-0.6	4.6	24.2	19.6	974	69.9	yellow soil
	Linshang Zhenglujiao	ZB2	Tianba Village Tianba Luding County Sichuan Province China	2050	2337	1646	17.5	-1.6	2.1	25.1	20.1	872	73.5	dark brown soil
	Da Hongpao	ZB3	Shuitang Village (G 1) Zhenping Town Songpan County Sichuan Province China	2440	2015	1916	14.3	-8.6	-2.6	22.0	16.1	816	67.5	brown soil
	Da Hongpao	ZB3	Shuitang Village (G 2) Zhenping Town Songpan County Sichuan Province China	2440	1053	1919	13.9	-9.2	-3.0	21.5	15.4	822	67.8	brown soil
	Da Hongpao	ZB3	Suoqiao Village (G 2) Yanmen Town Wenchuan County Sichuan Province China	1700	3308	1339	16.8	-3.6	1.1	24.8	19.8	830	76.7	cinnamon soil
	Da Hongpao	ZB3	Xinmin Village (G 2) Zhenping Town Songpan County Sichuan Province China	2307	1981	1920	14.6	-7.9	-2.1	22.4	16.6	815	68.0	brown soil
	Da Hongpao	ZB3	Xinmin Village (G 3) Zhenping Town Songpan County Sichuan Province China	2309	1975	1894	14.6	-8.0	-2.1	22.3	16.6	817	68.0	brown soil
	Da Hongpao	ZB3	Mayizu Town Jinyang County Sichuan Province China	1800	3622	1752	18.3	-0.3	3.0	24.6	20.1	1092	76.5	cinnamon-red soil
	Da Hongpao	ZB3	Taiping Village (G 1) Taiping Town Mao County Sichuan Province China	2200	2183	1930	15.6	-6.6	-1.1	23.4	17.8	812	68.2	brown soil
	Hanyuan Huajiao	ZB4	Xinli Village(G 4) Qingxi Town Hanyuan County Sichuan Province China	2200	2340	1556	16.6	-1.8	2.2	24.1	19.3	919	73.9	purple soil
	Hanyuan Huajiao	ZB4	Xinli Village(G 5) Qingxi Town Hanyuan County Sichuan Province China	1882	3112	1458	16.4	-2.1	1.9	23.8	18.9	924	75.2	purple soil
	Hanyuan Huajiao	ZB4	Shuangzhenshuangping Village (G 1) Qingxi Town Hanyuan County Sichuan Province China	1800	3305	1429	17.6	-0.4	3.2	25.4	20.7	907	75.0	purple soil
	Hanyuan Huajiao	ZB4	Guanhua Village (G 3) Yidong Town Hanyuan County Sichuan Province China	2400	1911	1618	15.5	-3.5	0.8	22.5	17.6	923	73.2	purple soil
	Hanyuan Huajiao	ZB4	Guanhua Village (G 4) Yidong Town Hanyuan County Sichuan Province China	2000	2254	1572	16.8	-1.8	2.1	24.2	19.4	905	73.6	purple soil
	Hanyuan Huajiao	ZB4	Dadi Village (G 5) Liyuan Town Hanyuan County Sichuan Province China	1824	3054	1581	17.5	-0.9	2.9	24.9	20.2	903	73.4	brown soil
	Hanyuan Huajiao	ZB4	Gaoqiao Village Sanjiao Yidong Town Hanyuan County Sichuan Province China	2092	1893	1712	15.0	-4.7	0.0	21.9	16.6	930	72.1	purple soil
