# Supplementary material for: The Chemical and Genetic Characteristics of Szechuan Pepper (Zanthoxylum bungeanum and Z. armatum) Cultivars and Their Suitable Habitat
Source: Front Plant Sci. 2016 Apr 19;7:467. doi: 10.3389/fpls.2016.00467 (PMC4835500; doi:10.3389/fpls.2016.00467)
Supplement: Supplementary file 4 [file Table4.PDF]

Supplementary Material

The chemical and genetic characteristics of Szechuan pepper cultivars and their suitable habitat

Li Xiang[1], Yue Liu[1] Caixiang Xie [2], Xiwen Li[1], Yadong Yu[1, 3], Meng Ye[3 *], Shilin Chen[1 *]

***Correspondence:**

Shilin Chen

slchen@icmm.ac.cn

Meng Ye

yemeng5581@163.com

Supplementary Table 4 Sequence characteristics of Szechuan peppers and its related *Zanthoxylum* species based on ITS2 sequence

Species	Length (bp)	G+C content (%)	Intraspecific distance (mean)	Interspecific distance (mean)
Z. bungeanum	224	66.5	0-0.0321 (0.0093)	0.0227-0.1340 (0.0754)
Z. armatum	227	70.7	0-0.0224 (0.0055)	0.0227-0.1335 (0.0645)
Z. nitidum	227	73.3	0.0134-0.0459 (0.0272)	0.0461-0.1493 (0.0849)
Z. myriacanthum	224	72.3	0	0.0414-0.1327 (0.1111)
Z. ovalifolium	224	69.5	0-0.0045 (0.0022)	0.0756-0.1493 (0.1127)
Z. piasezkii	224	68.8	0	0.0227-0.1278 (0.0516)
Z. schinifolium	224	71	0	0.0414-0.1335 (0.1238)
Z. piperitum	222	66.7	0	0.0373-0.1236 (0.0563)
Z. dissitum	224	73.8	0-0.0090 (0.0060)	0.0560-0.1236 (0.0969)
