# Supplementary material for: The Chemical and Genetic Characteristics of Szechuan Pepper (Zanthoxylum bungeanum and Z. armatum) Cultivars and Their Suitable Habitat
Source: Front Plant Sci. 2016 Apr 19;7:467. doi: 10.3389/fpls.2016.00467 (PMC4835500; doi:10.3389/fpls.2016.00467)
Supplement: Supplementary file 5 [file Table5.PDF]

Supplementary Material

The chemical and genetic characteristics of Szechuan pepper cultivars and their suitable habitat

Li Xiang[1], Yue Liu[1] Caixiang Xie [2], Xiwen Li[1], Yadong Yu[1, 3], Meng Ye[3 *], Shilin Chen[1 *]

***Correspondence:**

Shilin Chen

slchen@icmm.ac.cn

Meng Ye

yemeng5581@163.com

Supplementary Table 5 Sequence characteristics of 8 cultivars of Szechuan peppers based on ITS2 sequence

Species	Cultivars	Length (bp)	G+C content (%)	Intraspecific distance (mean)	Interspecific distance (mean)
Z. armatum	ZA1	227	70.5	0-0.0089(0.0044)	0-0.0446 (0.0337)
	ZA2	227	70.9	0	0-0.0446 (0.0371)
Z. bungeanum	ZB1	224	66.5	0	0-0.0417 (0.0139)
	ZB2	224	66.5	0	0-0.0417 (0.0147)
	ZB3	224	66.4	0-0.0320 (0.0121)	0-0.0446 (0.0277)
	ZB4	224	66.5	0	0-0.0417 (0.0126)
