# Supplementary material for: The Chemical and Genetic Characteristics of Szechuan Pepper (Zanthoxylum bungeanum and Z. armatum) Cultivars and Their Suitable Habitat
Source: Front Plant Sci. 2016 Apr 19;7:467. doi: 10.3389/fpls.2016.00467 (PMC4835500; doi:10.3389/fpls.2016.00467)
Supplement: Supplementary file 6 [file Table6.PDF]

Supplementary Material

The chemical and genetic characteristics of Szechuan pepper cultivars and their suitable habitat

Li Xiang[1], Yue Liu[1] Caixiang Xie [2], Xiwen Li[1], Yadong Yu[1, 3], Meng Ye[3 *], Shilin Chen[1 *]

***Correspondence:**

Shilin Chen

slchen@icmm.ac.cn

Meng Ye

yemeng5581@163.com

Supplementary Table 6A Major cultivars regions in China (SI 95%～100%, the cultivars belong to *Z. armatum*)

Province	Jinyangqing Huajiao (ZA1)			Tengjiao (ZA2)		
	City/County	Suitable producing area/km^2	Proportion of suitable producing area/%	City/country	Suitable producing area/km^2	Proportion of suitable producing area/%
Chongqing	12	670.39	1.52	11	204.78	2.1
Yunnan	20	1601.93	2.59	3	271.73	13.72
Sichuan	20	1151.59	2.63	52	10034.62	11.43
Hubei	1	28.51	0.66	—	—	—
Guizhou	12	1031.42	3.24	—	—	—
Total	65	4483.85	2.49	66	10511.13	10.23

Supplementary Table 6B Major cultivars regions in China (SI 95%～100%, the cultivars belong to *Z. bungeanum*)

Province	Yuexigong Jiao (ZB1)			Lingshan Zhenglujiao (ZB2)			Da Hongpao (ZB3)			Hanyuan Huajiao (ZB4)		
	City/County	Suitable producing area/km^2	Proportion of suitable producing area/%	City/country	Suitable producing area/km^2	Proportion of suitable producing area/%	City/country	Suitable producing area/km^2	Proportion of suitable producing area/%	City/country	Suitable producing area/km^2	Proportion of suitable producing area/%
Gansu	—	—	—	4	483.29	1.97	12	6474.45	9.22	3	200.78	1.49
Guizhou	1	173.53	3.02	1	449.37	7.84	1	310.02	2.7	—	—	—
Henan	—	—	—	—	—	—	3	10.51	0.08	—	—	—
Hubei	—	—	—	10	746.15	1.94	14	1919.45	2.92	2	60.65	0.95
Shaanxi	—	—	—	22	2900.77	2.88	40	8527.15	3.95	10	408.98	1.22
Sichuan	19	1594.13	2.51	61	31817.12	10.23	60	22412.21	7.24	45	6630.05	4.39
Tibet	—	—	—	4	2064.71	1.09	6	3860.93	1.29	3	184.18	0.19
Yunnan	14	1655.78	4.87	54	44480.38	17.63	31	21643.03	10.3	14	7194.35	9.93
Chongqing	—	—	—	6	2451.42	10.12	5	1750.34	5.24	2	107.55	1.58
Total	34	3423.44	3.26	162	85393.21	10.58	172	66908.09	6.3	79	14786.54	4.59
