# Supplementary material for: The Chemical and Genetic Characteristics of Szechuan Pepper (Zanthoxylum bungeanum and Z. armatum) Cultivars and Their Suitable Habitat
Source: Front Plant Sci. 2016 Apr 19;7:467. doi: 10.3389/fpls.2016.00467 (PMC4835500; doi:10.3389/fpls.2016.00467)
Supplement: Supplementary file 8 [file Image2.PDF]

Supplementary Material

The chemical and genetic characteristics of Szechuan pepper cultivars and their suitable habitat

Li Xiang[1], Yue Liu[1] Caixiang Xie [2], Xiwen Li[1], Yadong Yu[1, 3], Meng Ye[3 *], Shilin Chen[1 *]

***Correspondence:**

Shilin Chen

slchen@icmm.ac.cn

Meng Ye

yemeng5581@163.com

Species	GenBank No.	15	20	25	26	27	28	29	30	31	32	38	39	45	47	70	74	75	76	77	83	86	93	98	99	104	108	113	117	122	123	128	138	139	145	154	156	157	159	163	166	168	170	175	176	177	178	179	181	183	184	185	186	192	193	194	200	203	206	208	213	216	217	218	220	228	229
Z. bungeanum	KP642548	A	A	C	T	-	-	-	C	C	G	C	T	T	C	C	C	C	C	C	C	C	A	T	T	-	T	A	G	G	C	A	A	A	A	G	T	A	C	T	T	-	C	C	C	C	C	T	T	T	C	G	G	A	T	A	T	A	T	T	A	C	A	-	T	T	T
	KP642577	.	.	.	.	-	-	-	.	.	.	.	.	.	.	.	.	.	.	.	.	T	.	.	.	-	.	.	T	.	T	.	.	.	.	.	.	C	.	.	.	-	T	.	.	.	.	.	.	.	.	.	.	.	.	.	.	.	.	.	.	.	.	-	C	.	C
	KP642578	.	.	.	.	-	-	-	.	.	.	.	.	.	.	.	.	.	.	.	.	.	.	.	.	-	.	.	T	.	T	.	.	.	.	.	.	C	.	.	.	-	.	.	.	.	.	.	.	.	.	.	.	.	.	.	.	.	.	.	.	.	.	-	C	.	C
	KP642579	.	.	.	.	-	-	-	.	.	.	.	.	.	.	.	.	.	.	.	.	.	.	.	.	-	.	.	.	.	T	.	.	.	.	.	.	C	.	.	.	-	T	.	.	.	.	.	.	.	.	.	.	.	.	.	.	.	.	.	.	.	.	-	C	.	C
	KP642589	.	.	.	.	-	-	-	.	.	.	.	.	.	.	.	.	.	.	.	.	T	.	.	.	-	.	.	.	.	T	.	.	.	.	.	.	C	.	.	.	-	T	.	.	.	.	.	.	.	.	.	.	.	.	.	.	.	.	.	.	.	.	-	C	.	C
	KP642597	.	.	.	.	-	-	-	.	.	.	.	.	.	.	.	.	.	.	.	.	.	.	.	.	-	.	.	T	.	T	.	.	.	.	.	.	C	.	.	.	-	T	.	.	.	.	.	.	.	.	.	.	.	.	.	.	.	.	.	.	.	.	-	C	.	C
	KP642598	.	.	.	.	-	-	-	.	.	.	.	.	.	.	.	.	.	.	.	.	.	.	.	.	-	.	.	.	.	.	.	.	.	.	.	.	C	.	.	.	-	.	.	.	.	.	.	.	.	.	.	.	.	.	.	.	.	.	.	.	.	.	-	C	.	C
	KP642604	.	.	.	.	-	-	-	.	.	.	.	.	.	.	.	.	.	.	T	.	.	.	.	.	-	.	.	.	.	.	.	.	.	.	.	.	C	.	.	.	-	.	.	.	.	.	.	.	.	.	.	.	.	.	.	.	.	.	.	.	.	.	-	.	.	.
	KP642605	.	.	.	.	-	-	-	.	.	.	.	.	.	.	.	.	.	.	.	.	T	.	.	.	-	.	.	.	.	.	.	.	.	.	.	.	C	.	.	.	-	.	.	.	.	.	.	.	.	.	.	.	.	.	.	.	.	.	.	.	.	.	-	C	.	.
Z. nitidum	KP642614	.	.	.	.	C	C	C	.	.	.	.	C	.	.	.	.	.	.	.	.	.	-	C	G	C	C	.	.	.	.	.	.	.	G	.	.	C	.	.	.	-	T	.	G	.	.	.	.	C	.	.	.	.	G	G	C	C	G	.	G	.	G	-	C	.	C
	KP642615	.	.	.	.	-	C	C	.	.	.	.	C	.	.	.	.	.	.	.	.	.	-	C	C	C	C	.	.	.	.	.	.	.	G	.	.	C	.	.	.	-	T	.	G	.	.	.	.	C	.	.	.	.	G	G	C	C	G	.	T	.	G	-	C	C	C
	KP642616	.	G	.	.	-	C	C	.	.	.	.	C	.	.	.	.	.	.	.	.	.	-	C	A	C	C	.	.	.	.	.	.	.	G	.	.	C	.	.	.	-	T	.	G	.	A	.	.	C	.	.	.	.	G	G	C	C	G	.	G	T	G	-	C	C	C
	KP642617	.	.	.	.	-	C	C	.	.	.	.	C	.	.	.	.	.	.	.	.	.	-	.	C	-	C	.	.	.	.	.	.	.	.	.	.	C	.	.	.	-	T	.	G	.	.	.	A	C	.	.	.	.	G	G	C	C	.	.	G	.	.	-	C	.	C
Z. armatum	KP642516	.	.	.	.	-	C	-	.	G	.	.	.	.	.	.	.	.	.	.	.	.	.	C	.	-	C	.	.	.	.	.	.	.	.	.	.	C	.	.	.	C	.	.	.	.	.	.	.	C	.	.	.	.	.	G	C	.	C	.	.	.	.	G	C	.	C
	KP642517	.	.	.	.	-	C	-	.	G	.	.	.	.	.	.	.	.	.	.	.	.	.	.	.	-	C	.	.	.	.	.	.	.	.	.	.	C	.	.	.	C	.	.	.	.	.	.	.	.	.	.	.	.	.	G	C	.	C	.	.	.	.	G	C	.	C
	KP642527	.	.	.	.	-	C	-	.	G	.	.	.	.	.	.	.	.	.	.	.	.	.	C	.	-	C	.	.	.	.	T	.	.	.	.	.	C	.	.	.	C	.	.	.	.	.	.	.	C	.	.	.	.	.	G	C	.	C	.	.	.	.	G	C	.	C
	KP642531	.	.	.	.	-	C	-	.	G	.	.	.	.	.	.	.	.	.	.	.	.	.	C	.	-	C	.	.	.	.	.	.	.	.	.	.	C	.	.	.	C	.	.	.	.	.	.	.	.	.	.	.	.	.	G	C	.	C	.	.	.	.	G	C	.	C
	KP642533	.	.	.	.	-	C	-	.	G	.	.	.	.	.	.	.	.	.	.	.	.	.	.	.	-	C	.	.	.	.	.	.	.	.	.	.	C	.	.	.	C	.	.	.	.	.	.	.	.	.	.	.	.	.	G	C	.	C	.	.	.	.	G	C	.	C
	KP642541	.	.	.	.	-	C	-	.	G	.	.	.	.	.	T	.	.	.	.	.	.	.	C	.	-	C	.	.	.	.	T	.	.	.	.	.	C	.	.	.	C	.	.	.	.	.	.	.	C	T	.	.	.	.	G	C	.	C	.	.	.	.	G	C	.	C
	KP642547	.	.	.	.	-	C	-	.	G	.	.	.	.	.	T	.	.	.	.	.	.	.	C	.	-	C	.	.	.	.	T	.	.	.	.	.	C	.	.	.	C	.	.	.	.	.	.	.	C	.	.	.	.	.	G	C	.	C	.	.	.	.	G	C	.	C
Z. dissitum	KP642607	.	.	.	G	-	C	-	.	.	.	.	C	.	A	.	.	.	.	.	.	.	.	C	C	-	C	G	.	.	.	.	.	.	.	.	.	C	T	G	C	-	.	T	A	T	.	C	.	C	.	.	.	.	G	G	.	C	.	C	G	.	G	-	C	.	C
	KP642608	.	.	.	G	-	C	-	.	.	.	.	C	.	A	.	.	.	.	.	.	.	.	C	C	-	C	G	.	.	.	.	.	.	.	.	.	C	.	G	C	-	.	T	.	T	.	C	.	C	.	.	.	.	G	G	.	C	.	C	G	.	G	-	C	.	C
Z. myriacanthum	KP642610	G	.	.	G	-	C	-	.	.	.	T	.	C	A	.	A	.	.	.	.	T	.	A	C	-	C	.	.	.	.	.	C	.	.	.	.	C	.	G	.	-	.	T	.	T	.	C	.	C	.	.	.	G	A	G	.	C	.	C	G	.	G	-	C	.	C
Z. ovalifolium var. *spinifolium*	KP642636	.	.	.	G	-	C	-	T	A	C	.	.	.	.	A	.	.	T	.	T	T	.	.	C	-	C	.	.	.	.	.	.	G	.	.	.	C	.	G	.	-	.	T	.	T	.	C	.	C	.	C	.	.	G	G	.	C	.	C	.	.	G	-	.	.	C
Z. ovalifolium	KP642618	.	.	.	G	-	C	-	T	A	C	.	.	.	.	A	.	.	T	.	T	T	.	.	C	-	C	.	.	T	.	.	.	G	.	.	.	C	.	G	.	-	.	T	.	T	.	C	.	C	.	C	.	.	G	G	.	C	.	C	.	.	G	-	.	.	C
Z. piasezkii	KP642620	.	.	.	.	-	-	-	.	T	.	.	.	.	.	.	.	.	.	.	.	.	.	C	.	-	.	.	.	.	.	.	.	.	.	.	.	C	.	.	.	-	.	.	.	.	.	.	.	.	.	.	.	.	.	G	.	.	C	.	G	.	.	-	A	.	C
Z. schinifolium	KP642624	G	.	.	G	-	C	-	.	.	.	T	.	C	A	.	.	.	.	.	.	T	.	.	C	-	C	.	.	.	.	.	.	.	.	A	A	C	.	G	.	-	.	T	.	T	.	C	.	.	.	.	A	.	G	G	.	C	.	C	G	.	G	-	C	.	C
Z. piperitum	KP642622	.	.	T	C	-	C	-	-	-	.	.	.	.	.	.	.	T	T	A	.	T	.	.	.	-	.	.	.	.	.	.	.	.	.	.	.	C	.	.	.	-	.	.	.	.	.	.	.	A	.	.	.	.	.	G	.	.	.	.	.	.	.	-	C	.	C

Supplementary Figure 2 Variable sites and insertions/deletions for Szechuan peppers and related *Zanthoxylum* species based on ITS2 sequences. The specific variable sites and insertions/deletions are highlighted.
